# Mapping Epileptic Activity: Sources or Networks for the Clinicians?

**DOI:** 10.3389/fneur.2014.00218

**Published:** 2014-11-05

**Authors:** Francesca Pittau, Pierre Mégevand, Laurent Sheybani, Eugenio Abela, Frédéric Grouiller, Laurent Spinelli, Christoph M. Michel, Margitta Seeck, Serge Vulliemoz

**Affiliations:** ^1^EEG and Epilepsy Unit, Neurology Department, University Hospitals and Faculty of Medicine of Geneva, Geneva, Switzerland; ^2^Laboratory for Multimodal Human Brain Mapping, Hofstra North Shore LIJ School of Medicine, Manhasset, NY, USA; ^3^Functional Brain Mapping Laboratory, Department of Fundamental Neurosciences, University of Geneva, Geneva, Switzerland; ^4^Support Center of Advanced Neuroimaging (SCAN), Institute for Diagnostic and Interventional Neuroradiology, University Hospital Inselspital, Bern, Switzerland; ^5^Radiology Department, University Hospitals and Faculty of Medicine of Geneva, Geneva, Switzerland

**Keywords:** connectivity, resting-state network, epilepsy, animal model, EEG, fMRI, MEG, intracranial EEG

## Abstract

Epileptic seizures of focal origin are classically considered to arise from a focal epileptogenic zone and then spread to other brain regions. This is a key concept for semiological electro-clinical correlations, localization of relevant structural lesions, and selection of patients for epilepsy surgery. Recent development in neuro-imaging and electro-physiology and combinations, thereof, have been validated as contributory tools for focus localization. In parallel, these techniques have revealed that widespread networks of brain regions, rather than a single epileptogenic region, are implicated in focal epileptic activity. Sophisticated multimodal imaging and analysis strategies of brain connectivity patterns have been developed to characterize the spatio-temporal relationships within these networks by combining the strength of both techniques to optimize spatial and temporal resolution with whole-brain coverage and directional connectivity. In this paper, we review the potential clinical contribution of these functional mapping techniques as well as invasive electrophysiology in human beings and animal models for characterizing network connectivity.

## Introduction

Epilepsy is one of the most frequent chronic neurological disorder, with an incidence of 50/100,000/year and a prevalence of 0.5–1% (1, 2). One third of these patients are drug resistant (3). Focal seizures are classically considered to be caused by an abnormal neuro-electrical activity of a focal epileptogenic zone and a subsequent spreading to other brain regions. This concept is intimately linked to the correlation between ictal signs and symptoms, electro-physiological activity, and structural lesion [anatomo-electro-clinical correlation (4)]. Furthermore, this hypothesis is crucial to select drug-resistant focal epilepsy patients for surgery, a widely accepted effective therapy (5, 6). The aim of epilepsy surgery is to remove the epileptogenic zone with the preservation of the eloquent areas (7).

Recent progress in neuro-imaging and electro-physiology suggests that focal seizures and focal epilepsies are actually related to an abnormal function of a network of cortical and subcortical brain structures rather than to a single epileptogenic region (8–14). The occurrence of epileptic activity is due to the abnormal neuronal activity of these connected regions and abnormal interactions between them (epileptic network). This conceptual shift is reflected in the new terminology proposal for seizures and epilepsies of the International League against Epilepsy, which proposes “*focal*” as indicating seizures arising primarily “within networks limited to one hemisphere and that may be discrete or more widely distributed” (15). Generalized seizures are considered as “originating within and rapidly engaging, bilaterally distributed networks” of cortical and subcortical regions. Inside these networks, some brain regions are responsible for seizure initiation and propagation, whereas other nodes are more remotely involved, their activity modulating, or being modulated by the epileptic discharge.

There is increasing evidence that epileptic activity strongly interacts with physiological brain networks, notably the so-called “resting-state networks” (RSNs) (8, 16). A RSN is a set of brain regions that shows temporal correlations in their activity (as measured by hemodynamic or electrical signals) and that are functionally related. They are observed during rest but correspond to the networks revealed in different behavioral and cognitive task (e.g., attention, vision, etc.). This has led to the new concept that the apparently resting spontaneous brain activity shows continuous interaction among brain networks responsible for various classes of sensory/behavioral functions (17). RSNs are highly organized in space, reproducible from subject to subject, and differ with aging and between genders (18).

In this paper, we review the converging evidence from different brain mapping techniques in human and animal models that epilepsy is related to the dysfunction of a large-scale brain networks, with alterations of physiological brain networks. We will particularly focus on the clinical impact of this new view of epilepsy as a network disease.

## Methods

An electronic literature search was conducted for articles on this topic regarding human and animal subjects. Sources searched included PubMed and relevant books. Words used in the search included the text words and subject headings of epilep*, functional connect*, resting-state functional network*, temporal epilepsy, extra-temporal epilepsy, electroencephalogram (or EEG), simultaneous functional MRI (fMRI) and EEG (or EEG-fMRI), electric and magnetic source imaging (or MSI, ESI), intracranial EEG (or iEEG or sEEG), cortico-cortical evoked potential, and single-pulse electrical stimulation. The words were searched independently and in combination. For each citation considered, the abstract was read (when available), and articles were excluded if they were outside the scope of the review. Studies published only in abstract form, letters, and technical reports were excluded. The bibliography of each of the retrieved papers was examined to identify relevant references that could have been missed by electronic search. The findings were described taking into account the limit of words and the critical insight of the authors.

## How to Measure Resting-State Networks? Functional Connectivity

Functional interactions between brain regions activity, can be characterized in several ways. On the one hand, functional connectivity (FC), the most widely used metrics, measures the statistical dependency between different signals obtained by correlation analysis. However, such strategy does not account for the direction of the information flow and cannot therefore infer causality relationships. On the other hand, effective or directed connectivity investigates directional relationships and aims at describing causal influences. Effective connectivity can be investigated using model-driven techniques such as structural equation modeling (19) and dynamic causal modeling (DCM) (20), data-driven techniques such as Granger-causal modeling (21), or by recording the response of remote areas to focal stimulation of a given brain region [cortico-cortical evoked potentials (22)]. Connectivity studies can be applied among a set of predefined relevant brain regions selected by the investigator, between one seed region and the rest of the brain or at the whole-brain scale, using the spatial resolution of the recording technique. A detailed description of the various approaches used for measuring connectivity is beyond the scope of this review and the reader is referred to studies comparing various approaches to better understand the specific limitations of each technique (23–25). The results obtained by such connectivity analysis between all pairs of brain regions can be represented in so-called connectivity matrices. Graph topological analysis is then increasingly applied to reduce the complexity of the data and extract meaningful characteristics of the networks (26).

### Blood oxygen level dependent signal and physiological resting-state networks

The concept of brain networks originated, and has largely benefited, from the use of resting-state fMRI. fMRI detects blood oxygen level dependent (BOLD) signal change reflecting metabolically active brain areas not only in relation to a specific physiologic or pathologic event (27) but also in resting-state (RS) condition (resting-state-fMRI or RS-fMRI).

Biswal and colleagues demonstrated for the first time (1995) that brain regions that are functionally related, show temporal correlations in the low frequency component of the BOLD signal. In other words, fMRI FC detects zones that exhibit correlated BOLD fluctuations and, as a result, belong to the same functional network (28). Studies in monkeys (29) and in human beings (30) suggest that FC is related to neuronal processes.

Functional connectivity can be measured while the subject is performing a behavioral and cognitive task (task-related FC), or while the subject is not performing any specific task (RS-FC). The RSN that is mainly activated in condition of resting wakefulness and deactivated in task performing is called default-mode network (DMN) (31). This physiological RSN is involved in self-referential thoughts and consciousness (32, 33). The concept of “resting” is debatable. Usually, subjects are instructed to lie down in the scanner with the eyes closed, and are invited to not sleep.

Different methods have been developed to extract RSNs, some requiring an “*a priori* hypothesis,” like seed-based approach (34), other do not [i.e., independent component analysis (35), or bootstrap analysis (36)]. The description of the methodological aspects is outside the scope of this review. Other papers can be consulted (14, 37, 38).

### EEG/MEG and physiological resting-state networks

Functional connectivity algorithms similar to those used for fMRI BOLD signals can be applied to MEG or EEG current-density estimations in the source space, revealing brain areas that are synchronized in specific frequency bands. As with fMRI, such analysis can be applied to task-related (39), as well as to spontaneous resting-state activity (40, 41). The unique advantage of EEG/MEG connectivity analysis is the high temporal resolution that allows studying fast fluctuations within large-scale network interactions and fast switches between resting-state networks.

FC analysis of EEG/MEG considers the time-course of electro-magnetic signals and looks at correlations of oscillating networks (42). Beyond this view of temporal oscillations, EEG recordings can be considered as time-series of scalp potential maps that vary across time with the temporal resolution in the order of milliseconds (43). Several studies have shown that spontaneous EEG signals can be explained by the alternation of periods of stable topography, lasting almost 100 ms, very reproducible across subjects, and modifiable by neurological (44) or psychiatric impairment (45). These periods are called microstates and can be identified throughout an individual’s life (46) suggesting that they might be mediated by predetermined anatomical connections. During rest, four different microstates are consistently observed, and they can be considered as “basic building blocks” of spontaneous mental activities (47). A recent review on this topic is available (48).

It has been shown (49) that the temporal dynamic of EEG microstates have hemodynamic correlates that can be measured with EEG-fMRI and that each physiological microstate map corresponds to one of the well-described BOLD RS network. Such clear correlates between EEG and BOLD are less well found when looking at classical power fluctuations in specific EEG frequency bands (50). This finding strongly suggests that the EEG microstates can be the candidates for the electro-physiological signatures of fMRI RSNs. Scale-invariance of the alternation between microstates has been demonstrated to be the base of this coupling over such a wide temporal scale (51).

## Evidence for Brain Networks Involved in Epileptic Activity

As described above, FC at the whole-brain level can be studied with EEG, MEG, fMRI, iEEG, or the combination of these techniques. They have been applied to patients with focal or generalized epilepsy to characterize spatial and temporal properties of epileptic networks.

### EEG and MEG-based connectivity in epilepsy

EEG and MEG are appealing non-invasive techniques for estimating brain connectivity in epilepsy because they measure neuro-electrical activity more directly than fMRI and can potentially offer a higher temporal resolution.

Several studies using concordance with intracranial recordings or post-operative outcome have established that electric and magnetic source imaging (ESI, MSI) are reliable techniques for estimating the localization of the cortical generators of epileptic activity (52–55) and these techniques now offer a much more convincing strategy to investigate connectivity directly between the activity of cortical regions. Therefore, both ESI and MSI studies will be discussed together hereunder. Studies using connectivity analysis in the sensor space are not discussed here because of their severe limitations of interpretation due to important caveats related to sensor cross-talk, volume conduction, and reference choice of the electromagnetic signals (56). The projection of the signal in source space requires the selection of a head model describing the propagation of the electromagnetic signal (forward problem) and an inverse solution (estimating the cortical activity from the EEG/MEG recording, inverse problem) (48, 57, 58). A variety of head models exists, from template averaged normal brain to highly sophisticated realistic models based on individual anatomy, and they have been used in epilepsy imaging and cognitive neurosciences. Validation in patients with invasive EEG or surgical resection showed that the individual anatomy was important for the localization accuracy (54), but that the performance of highly sophisticated models did not outperform less computer-intensive models also based on individual anatomy, as these were disturbed by the presence of brain lesions in patients with epilepsy (59). Regarding inverse solutions, dipole models consider a single or a few equivalent dipole(s) as sources of the EEG/MEG signals of sources distributed in the whole cortex (48). While both approaches might yield complimentary results for localizing epileptic sources (60), distributed sources are best suited to the study of connectivity between cortical patches at a large brain scale.

The analysis of interictal epileptic discharges has principally aimed at localizing epileptic generators in the context of pre-surgical evaluations rather than studying large brain networks. Case reports or small MEG series showed promising results for the localizing value of the regions with high information outflow, estimated by connectivity analysis (61–63). In addition, based on development in cognitive neurosciences, the background activity measured by MEG and EEG in the classical frequency bands has also been used as a substrate to estimate abnormal connectivity in patients with epilepsy and correlate it with clinical variables. In patients with brain tumors, increased theta-band connectivity and more profound network alterations were associated with a higher number of epileptic seizures (64) and there is higher post-operative network improvement in patients who become seizure free (65).

In generalized epilepsies, connectivity studies have highlighted a network of hyperconnected anterior regions in photosensitive patients (66). Network analysis using graph theory in five patients with absence epilepsy showed a build-up of connectivity changes occurring before the onset of generalized spike-wave discharges (67). This shows the potential of such a technique for our understanding of the large-scale brain networks underlying hyperexcitability and interictal to ictal transition. A similar approach has been applied to iEEG recordings of interictal to ictal transition in patients with focal cortical dysplasia (12).

Another study used co-occurrence of MEG interictal spikes to build graphs of connectivity between the estimated sources of these spikes. In seven patients also investigated with stereotactic iEEG, the connections revealed by MEG were confirmed by iEEG (68).

Similarly to fMRI studies, future work will need to distinguish between transient connectivity alterations related to interictal discharges, that are known to be associated with subtle cognitive impairment (69), and deeper connectivity changes based on background activity alterations. The tools are now available to benefit from the high temporal resolution of EEG/MEG to further investigate these issues and this field has recently attracted an intense interest. While MEG offers advantages over EEG for longitudinal studies of post-operative cases, due to its insensitivity to skull defects, the development of long-term high-density EEG system, its greater versatility compared to MEG and its potential combination with fMRI will be precious for recording seizures and exploring network changes leading to their initiation, spread, and termination.

### EEG-fMRI connectivity in epilepsy

**Figure 1:**
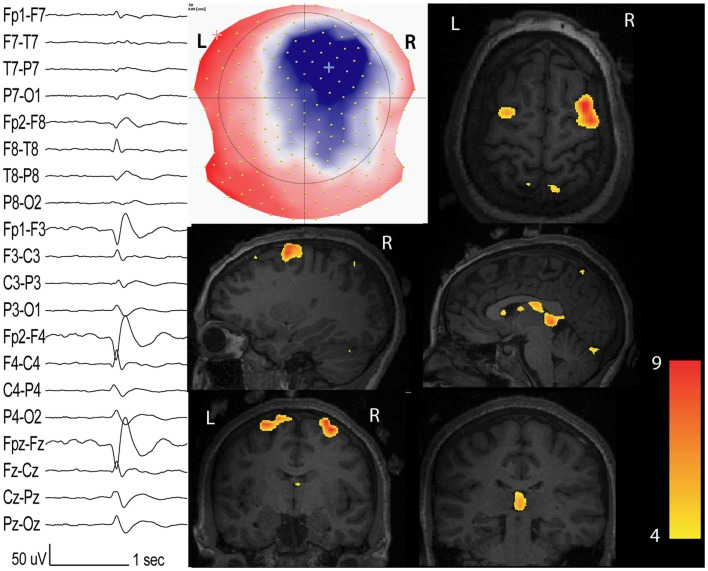
**Interictal network revealed by EEG-fMRI**. Patients with non-lesional right frontal epilepsy. Marked events inside the EEG-fMRI session: right frontal spikes with phase reversal at F4 (on the left: longitudinal bipolar montage from 204 channels EEG). On the middle: scalp voltage map of the spike (204 electrodes, viewed from the top) with the maximal right frontal negativity (blue). BOLD response (*t*-value = 4; *p* < 0.05 corrected for family-wise-error) has maximal activation in the spike topography but other clusters with inferior statistical values are present in the contra-lateral homologous region and in the thalamus.

Simultaneous EEG and fMRI (EEG-fMRI) detects hemodynamic changes in the brain related to events of interest identified in the EEG (70). Combining high temporal resolution of EEG signal with high spatial resolution of BOLD images, EEG-fMRI has been shown to be useful to characterize various forms of focal and generalized epileptic abnormalities (hereunder called “spikes” for practical reasons) (71). EEG-fMRI helps to localize epileptic focus in patients with drug-resistant focal epilepsy candidate for surgery (72, 73). From the first publications (74, 75), EEG-fMRI has demonstrated that BOLD responses to a focal spike can be multifocal, also present at a distance from the presumed focus (Figure 1), corroborating the concept of epileptic network (9). Studying such networks can inform about patients’ prognosis after surgery. While focal responses predict a good post-operative outcome, diffuse results are associated with a poor outcome, probably reflecting that a larger network is involved in the epileptogenic zone (76, 77). Epileptic activity can also be detected in the absence of spikes and fMRI analysis based on EEG topography can reveal epileptogenic networks (78).

BOLD responses to a neural event are usually detected with a delay of 4–6 s (79). Nevertheless, hemodynamic changes to spikes can have different peak times (80), and can occur before the spike is visible on the scalp (81). Dynamic analysis of BOLD response (82, 83) can tell us which brain areas are first activated, by comparing early BOLD response vs. late BOLD response. However, this analysis does not address the concept of causality and the sluggishness and variability of BOLD responses prevent a more accurate investigation into the temporal dynamics and directionality of the connections (24). Causality within epileptic network can be addressed by effective connectivity approaches like Dynamic Causal Modeling (DCM) (37, 84).

**Figure 2:**
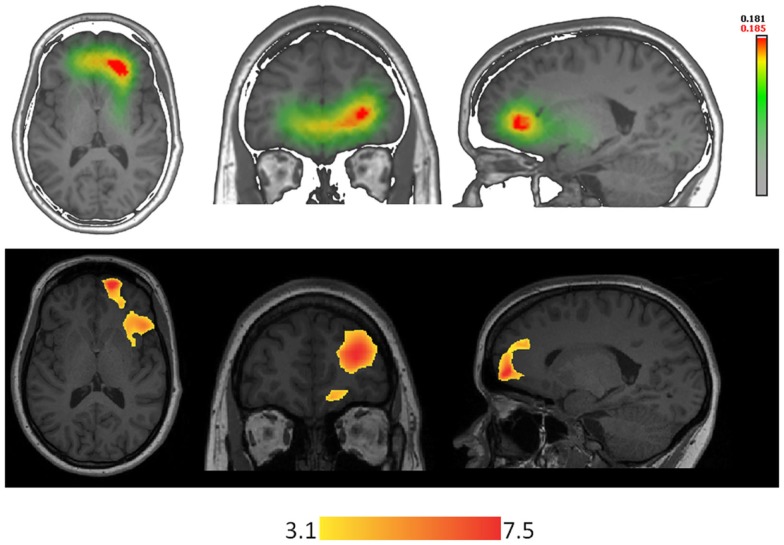
**Techniques using different types of signal are concordant in localizing the epileptic focus in a patient with right orbito-frontal focal cortical dysplasia**. On the top-line: ESI (256 electrodes, simplified realistic head model lSMAC, distributed inverse solution LORETA) performed on right frontal spikes (FP2-F8). On the bottom-line: EEG-fMRI performed on the same type of events recorded inside the scanner.

The combination of ESI with EEG-fMRI can offer complementary information for improving each single technique (Figure 2). Although EEG-fMRI and ESI measure different signals (hemodynamic the first, electrical the second), the concordance between ESI performed during fMRI recordings can allow distinguishing between hemodynamic changes related to spike onset vs. propagation, adding important temporal information to the limited fMRI temporal resolution (85, 86).

**Figure 3:**
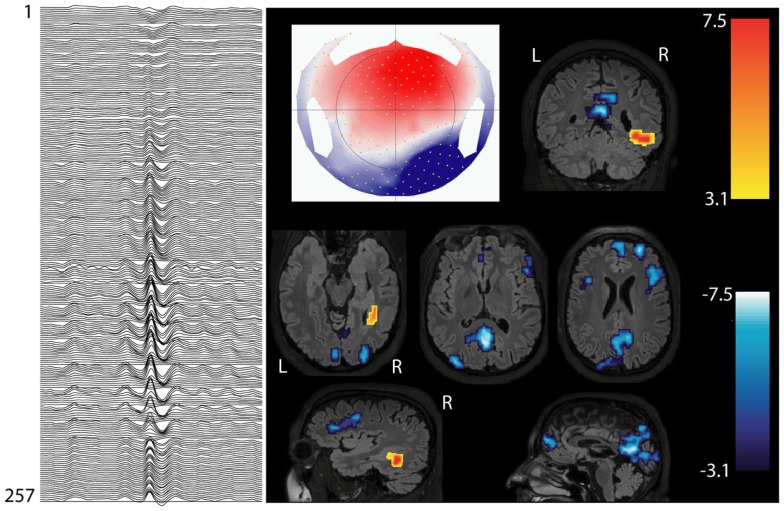
**Interictal involvement of DMN in focal epilepsy**. Patients with right hemispheric extended periventricular nodular heterotopia. Marked epileptic events inside the scanner: right posterior temporal spikes with phase reversal at P8 (on the left: 256 channel EEG; referential montage Fz-Cz). BOLD increase is concordant with the spike topography (topographic map on the middle), whereas BOLD decrease is present in the regions of default-mode network (DMN).

EEG-fMRI studies can give insights about epileptogenesis. Interictal spikes of different types of epilepsy (frontal, temporal, and posterior quadrant), are associated with deactivation in the precuneus and posterior cingulate cortex (10), regions involved in the DMN (Figure 3). Other physiological RSNs could be affected by spikes: this interaction and its clinical consequences need to be clarified in future studies. A common involvement of the cingulate gyri in temporal lobe and frontal lobe epilepsy was reported (10), probably resulting from rapid spread of epileptic activity originating from the temporal and frontal areas, which both involve the limbic system.

A specific area, localized in the medial orbito-frontal gyrus (piriform cortex), called “area tempestas”, seems to be involved in the genesis or propagation of epileptic activity (87, 88) in focal epilepsies. A DCM study supported the hypothesis of a causal link between hemodynamic changes in this structure and a specific type of reflex epilepsy, although in a single patient (89). Several other findings seem to corroborate the important role of the area tempestas: (i) its decrease in benzodiazepine receptor (87), (ii) its epileptogenic role in animal kindling models of temporal lobe epilepsy (TLE) (90–92), and (iii) its increase in gray matter volume in patient with frontal lobe epilepsy when compared to controls (93).

From a methodological point of view, multimodal combination between EEG/ESI, fMRI, and diffusion imaging tractography will allow exploring functional and structural connectivity at a finer spatio-temporal scale. Some initial small studies have highlighted the potential of these combinations (94–96).

#### fMRI and EEG-fMRI studies in focal epilepsy

Unfortunately, only few of the many studies on RS-FC have been done with the simultaneous recording of EEG. Spikes cause a transient cognitive impairment, by affecting, e.g., memory retrieval in rats (97), and memory maintenance and retrieval in human beings (69). Therefore, a more consistent use of simultaneous EEG while performing fMRI for RS or task-related studies in epileptic patients is needed to determine the influence of spikes on the determined BOLD networks. Indeed a study where EEG activity was monitored during a working memory-fMRI session (98) has shown that the task-related BOLD dramatically changes when spikes occur. Another advantage of the simultaneous recording of EEG in the scanner is that it allows monitoring the transition between different alertness states in order to assure that the subject is in RS and not in drowsiness/sleep state. A very recent review (99) has accurately discussed this issue and summarized the relevant studies.

##### Temporal lobe epilepsy

The majority of RS-fMRI studies in focal epilepsies have focused on TLE, which is the most common form of focal epilepsy in adults and offers the advantage of being one of the most homogeneous groups within the focal epilepsy syndromes.

Temporal lobe epilepsy has been the first epileptic syndrome to be considered as epileptogenic network (100) with relatively well characterized components encompassing different structures in the mesial temporal lobe (amygdala and hippocampus), adjacent cortex including enthorhinal cortex and lateral temporal cortex, and extra-temporal structures (i.e., thalamus and orbito-frontal cortex). fMRI connectivity studies (some with simultaneous EEG recording, others without) conducted by seeding the principal nodes of the mesial temporal network showed impaired connectivity with the other nodes of the network (101–103). Decreased connectivity is the most common finding among those studies. Nevertheless, there are reports of increased function of the unaffected hippocampus in patients with unilateral MTLE, both in the RS (104) and during task-related (105, 106) acquisitions. Morgan et al. (107) have shown that RS cross-hippocampal FC is disrupted at the beginning of the disease and then increases linearly with epilepsy duration after 10 years, suggesting that length of disease influences FC patterns. Bettus et al. (108) studied the electro-physiological correlates of BOLD signal fluctuations in structures exhibiting epileptiform discharges, by measuring correlations between intracerebral EEG and resting-state fMRI in five patients with TLE. They found an increase in connectivity measured from the intracerebral EEG but a decrease of connectivity measured from the BOLD signal in regions with epileptiform abnormalities relative to non-affected areas. This discrepancy, obtained by measuring connectivity of two signals of different nature (electrical and hemodynamic), demonstrates the challenge in interpreting connectivity changes. It could also suggest an alteration of neurovascular coupling in TLE.

In unilateral mesial TLE (MTLE), the affected amygdala and hippocampus (and to a lesser extent on the contra-lateral side) are less connected between them and also with other consistent RSNs, such as the mesolimbic and the DMN, suggesting that these functional interictal changes explain cognitive and psychiatric impairments often found in patients with this type of epilepsy (109). Several fMRI studies, with and without EEG, have shown an abnormal FC between physiological consistent RSNs [i.e., language (110)] and MTL structures.

*Default-mode network*. Laufs and colleagues (8) have shown that deactivation in DMN, involved in consciousness, is more frequent for spikes in patients with TLE than extra-TLE. Deactivation in the same regions in response to temporal spikes was also demonstrated by Kobayashi et al. (111) and by Fahoum et al. (10). Frings et al. (112) showed decreased DMN-hippocampus FC in MTLE patients compared to controls during an object-location memory task, underlying the importance of the intact connection between these structures to preserve memory. This concept was validated in post-surgical follow-up studies (see below). An impairment of the connections between DMN and MTL structures has been demonstrated also in RS with a seed-based fMRI analysis (113). The same group (114) combined fMRI RS-FC and diffusion tensor imaging, and showed that the decreased FC within the DMN in MTLE is correlated to abnormal structural connectivity. Although functional DMN connectivity is generally decreased in MTLE, few nodes can be hyperconnected and this may play a compensatory role for the loss of functional connections in other regions of the network (115). The same study, performed with an independent component analysis, has also shown distinct patterns of FC impairment with DMN between the left and right MTLE. The same difference has been further reported (116), suggesting that impaired cognition and memory in TLE may be different in right vs. left TLE. Morgan and colleagues (117) have identified a region in the ventral lateral nucleus of the right thalamus whose connectivity to the hippocampi separates left from right TLE subjects, suggesting that quantifying resting-state FC across this network may be a potential indicator of lateralization of TLE (useful step in the pre-surgical assessments).

Functional connectivity findings are related with clinical data: McCormick et al. (118) shows that MTLE patients with respect to controls have reduced connectivity from the posterior DMN to the epileptogenic hippocampus and increased DMN connectivity to the contra-lateral hippocampus. Stronger DMN connectivity to the epileptogenic hippocampus was associated with better pre-surgical memory and with greater postsurgical memory decline, whereas stronger DMN connectivity to the contra-lateral hippocampus was associated with less postsurgical memory decline. Following surgery, DMN connectivity to the remaining hippocampus increased from pre-surgical values and showed enhanced correlation with postsurgical memory function.

Hippocampi are considered by some authors as nodes of the DMN (119), but there is not unanimity on this interpretation (32, 120, 121). It is important to remember that all the regions of the brain can be functionally connected: a region belonging to a specific network (like the mesial temporal network) can belong also to a less specific network encompassing the previous one. This classification depends on how many different physiological RSNs are extracted from specific analyses: for instance, by extracting four physiological RSNs, the probability that the mesial temporal regions will be included in the DMN is higher than if the number of extracted network is higher (122).

*Mesolimbic network*. Patients with unilateral MTLE have important decreases of FC with the ventromesial limbic prefrontal regions and with the nucleus accumbens (109). These regions belong to a dopaminergic mesolimbic network, involved in long-term memory for novel events and reward (123). Hippocampus and amygdala have been often described as part of this network (124, 125), and several findings suggest that this network is affected in MTLE. The preferential seizure spread from mesial temporal lobes to mesial frontal lobes, especially the orbito-frontal cortex, has been demonstrated by ictal iEEG in patients with MTLE, suggesting that mesial orbito-frontal cortex is strongly affected by mesial temporal activity (126). On the other hand, dopaminergic alterations have been demonstrated in the pathophysiology of major depression, and dysfunctional activity of the mesolimbic dopaminergic system plays a crucial role in depressive behavior (127, 128). Structures belonging to mesolimbic network are functionally (129) and structurally (130) impaired in MTLE patients who have psychiatric impairments, such as anxiety/depression. A recent study (131) showed that the subgenual anterior cingulate cortex (mesolimbic network key-node) is affected only in MTLE patients that have primary affective disorders and not in those without such disorders and neither in controls. The same study confirms these FC findings with voxel-based morphometry and diffusion tensor imaging, corroborating the concept that the affective psychopathology often diagnosed in patients with MTLE has a neurobiological correlate. Antidepressant drugs, when effective, could modulate these connectivity impairments.

The amygdala has often been described as part of mesolimbic network and it is also involved in emotional processing of stimuli. Facial emotion recognition, particularly for “fear,” is impaired in patients with TLE, especially on the right hemisphere (132–135). Broicher and colleagues (136) showed through fearful-face fMRI-paradigm that the altered amygdala FC in TLE patients is strongly related to the poor performance in behavioral tests evaluating the theory of mind abilities (ability of decoding thoughts and behavior of other human beings). Another study with the same paradigm showed that, in right TLE patients, pre-operative right amygdala activation correlates with post-operative change of anxiety and depression scores [i.e., greater increases in anxiety and depression in patients with greater preoperative activation (137)]. This confirms that pre-surgical study of FC between TLE and other brain structures can help to predict post-surgery neuropsychological consequences.

*Attention network*. Several studies have shown that dorsal and ventral attention networks are functionally altered in MTLE, explaining why patients with this type of epilepsy have often worse performances than healthy controls (HC) in attention tasks. Cataldi et al. (138) have recently reviewed this topic.

##### Extra-temporal lobe epilepsy

Extra-temporal lobe epilepsies are characterized by a wide range of focus localization and etiology. For this reason, group studies with homogeneous clinical phenotype are difficult to achieve. This contrasts with the large body of group studies in MTLE, which take advantage from the frequent uniform pathology of atrophy and cell-loss in amygdala-hippocampus structures. A group-analysis EEG-fMRI study in different types of epilepsy (frontal, temporal, and posterior quadrant) showed that focal spikes are associated with networks of widespread metabolic changes, specific for each type of epilepsy (10).

Negishi et al. (139) revealed higher lateral pre-surgery FC maps in drug-resistant patients with good surgical outcome (seizure-free at 1-year) compared to those with poor outcome. A recent study on 23 patients with frontal lobe epilepsy used the same seed-FC approach (seed at maximal BOLD value of the spike-related activation map) (140), finding an increased FC in the neighborhood of the seed and a decrease in regions remote to the seed compared to controls. Patient-specific connectivity pattern was not significantly changed when comparing fMRI runs with spikes vs. fMRI without any spike detectable on the simultaneous EEG. Patients with drug-resistant frontal lobe epilepsy (141) recruit wider areas compared to controls when performing an fMRI memory encoding task paradigm, particularly in the contra-lateral frontal lobe, suggesting the presence of compensatory mechanisms to maintain memory function.

#### Generalized epilepsy

Different theories have been proposed about the patho-physiology of “generalized seizures”. Meeren et al. (142) reviewed this topic. All these theories consider cortex and thalamus as being involved in the generation of the typical 2.5–4 Hz generalized spike-wave discharges (GSWD) detected on scalp EEG, but it is still unclear, which of them is the “primum movens” (143). As discussed below, animal studies in genetic models of absence epilepsy are crucial to gain understanding of these conditions because no invasive validation can be contemplated in human beings. These animal studies suggest that GSWDs are triggered in a restricted cortical region (144–147).

Several EEG-fMRI studies showed that during short GSWD (16, 148–150) and absence (151–154), there is a characteristic pattern of subcortical (medio-dorsal thalamic and striatus) activation and cortical deactivation, especially in areas of the DMN. It has been hypothesized that the DMN deactivation is linked to reduced consciousness (i.e., absences) (16, 155, 156). A dynamic analysis study on 17 absences from nine patients with absence epilepsy and classical pattern of 3–4 Hz GSWDs (83) showed that BOLD responses were remarkably consistent in space and time across different absences of one patient, but were different from patient to patient. Furthermore, this study shows early frontal BOLD activations (specific for each patient), supporting the cortical focus theory. Another EEG-fMRI study on 11 children with absence seizures (157) revealed that the first brain zone showing BOLD increase was the parietal cortex, this activity starting 10 s before the onset of the discharge. Additionally, this study demonstrated the hemodynamic involvement of subcortical structures in GSWD, including the reticular structures of the brainstem. Focal cortical involvement before the onset of GSWD has been demonstrated also by a magnetoencephalography study in human beings (158) and a near-infrared spectroscopy study applied on the frontal cortex (159). An exhaustive review on focal abnormalities in idiopathic generalized epilepsy (IGE) has been recently published (160). All these findings support the conceptual transition from “primarily generalized epilepsy,” (implying that all brain regions simultaneously would generate GSWD) to seizures “originating within and rapidly engaging, bilaterally distributed networks” of cortical and subcortical regions (15).

Concerning the role of subcortical structures, in patients with IGE, it has been shown that both the anterior nucleus of thalamus (ANT) and the centromedian/parafascicular (Cm/Pf) nucleus (which provides diffuse inputs to the cortex) are activated during GSWD; the activity of the cortico-reticular Cm/Pf preceded that of the ANT, suggesting that the Cm/Pf is involved in GSWD initiation or early propagation, while the ANT in its maintenance (161).

**Figure 4:**
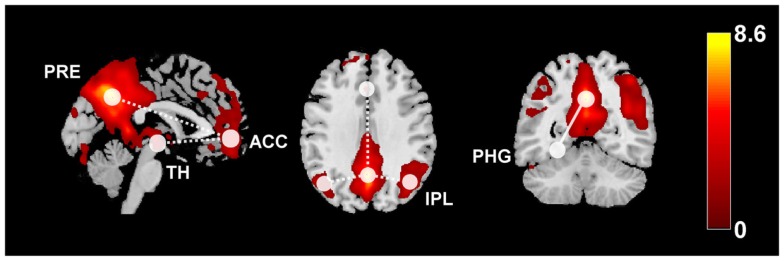
**This diagram summarizes the functional connectivity (FC) changes in patients with idiopathic generalized epilepsy compared to healthy controls**. The color map shows the default-mode network (z-scores) derived from independent component analysis or RS-fMRI data overlaid on a standard single-subject anatomy (Montreal Neurological Institute space). Widespread FC reductions were found within the DMN (dashed lines), as well as between anterior DMN and the thalamus. Increased FC related to increased disease duration has been observed between posterior DMN regions and the parahippocampal gyrus (solid line). ACC, anterior cingulate cortex, IPL, inferior parietal lobule, PRE, precuneus, PHG, parahippocampal gyrus, TH, thalamus.

Recent studies have used fMRI to investigate whether resting-state FC between thalamus, basal ganglia, and DMN areas is altered in patients with IGE, even during GSWD-free periods of brain activity (Figure 4). Wang et al. (162) used ICA to map RSNs in 16 patients with IGE and 16 HC. They found that the DMN had simultaneously reduced FC within the anterior cingulate cortex, but increased connectivity in the precuneus. Moreover, they found widespread connectivity reductions in prefrontal, sensorimotor, and even auditory cortices (162). Reduced resting-state FC between frontal areas and the rest of the DMN was later confirmed (163). An important question is whether these changes in DMN connectivity are meaningfully related to clinical information, e.g., disease duration or responsiveness to medication. Of note, in both aforementioned studies, there were significant correlations between RS-FC and disease duration: the reduction in connectivity was inversely correlated to disease duration, indicating that long-standing epilepsy leads to progressive disruption of DMN integration. Interestingly, a study of structural and FC in 26 IGE patients and HC, showed that the degree of coupling between functional and structural connectivity networks is decreased, and exhibited a negative correlation with epilepsy duration in patients (164).

Other RSNs can be affected in patients with IGE, reflecting specific cognitive impairment when compared to controls. A seed-based RS-FC study in 14 patients with IGE showed that attention network is impaired even in interictal periods, and that this impairment is related with the disease duration (165).

One study in 60 IGE patients specifically addressed the question whether pharmacoresistance was correlated with RS-FC changes in the DMN (166). DMN connectivity was reduced in the IGE group compared to HC, and the strongest decrease was found in those patients that were resistant to valproate. Finally, a recent study directly addressed RS-FC within the thalamo-cortical system (167), finding decreased RS-FC between thalamus and anterior DMN. Collectively, these studies suggest that there is a loss of functional integration in the thalamocortical and default-mode system of the brain in IGE, even outside the GSWD. Although small sample size and heterogeneous methodology limit “generalization,” the abnormal RS-FC patterns found in IGE so far could serve as endophenotypes of different IGE syndromes, and thus inform clinical diagnostics. Importantly, the confounding effect of anti-epileptic drug on dysconnectivity needs to be further elucidated.

The most frequent IGE syndrome is juvenile myoclonic epilepsy (JME), where seizures are characterized by myoclonic jerks of the upper limbs, often triggered by cognitive inputs. Several RS and task-related functional studies have shown an impairment of connectivity among supplementary motor area and the rest of the brain (168–170), suggesting that this structure may represent the anatomic basis for triggering motor seizures in JME. JME patients have often personality characteristics suggestive of a frontal lobe dysfunction (e.g., risk-taking behavior, dysexecutive syndrome). A task-related FC study in JME patients (171) shows that patients with ongoing seizures learn less from previous experiences compared to seizure-free patients and to controls.

#### Pediatric syndromes

Numerous EEG-fMRI studies have been conducted on pediatric syndromes [for review, see in Ref. (172)]. Several studies in Lennox–Gastaut syndrome (173–175) have shown hemodynamic involvement of brainstem, thalamus, and basal ganglia during paroxysmal fast activity and slow spike-and-wave discharges, underlying the importance of cortical–subcortical networks in Lennox–Gastaut syndrome. A group-analysis study in patients with myoclonic-astatic-epilepsy (176) showed that GSWD are related not only to a thalamo-cortical network (commonly found in IGE), but also to brain areas associated with motor function, suggesting that the involvement of these structures may predispose to the typical myoclonic jerks observed in this syndrome.

Concerning idiopathic focal epilepsies of childhood, these comprise a broad spectrum of phenotypes showing an overlap with each other, from benign childhood epilepsy with centro-temporal spikes (BECTS) to more severe seizure and cognitive disorders, like atypical benign partial epilepsy (ABPE), continuous spikes and waves during slow sleep (CSWS), and Landau-Kleffner syndrome. In patients with BECTS, EEG-fMRI studies have revealed focal spike-associated BOLD signal changes in the sensorimotor cortex, well corresponding to spikes localization, and typical seizure semiology (177–180). In patients with CSWS, a consistent neuronal network including both cortical and subcortical structures was described with positive BOLD signal changes in the perisylvian region, insula, cingulated cortex, and thalamus, and negative BOLD signal changes in the DMN areas and caudate nucleus (181). Source analysis of the simultaneously recorded EEG in these patients allowed differentiating initiation from propagation of epileptic activity in these common networks. The importance of assessing sleep state when studying networks is given by the report of a patient, whose centro-temporal spikes were recorded during wakefulness and sleep. BOLD response during wakefulness showed a focal activation concordant with the spike topography, whereas BOLD response to the same event during sleep showed the involvement of a thalamic–perisylvian neural network similar to the one previously observed in patients with CSWS, suggesting a common sleep-related network dysfunction even in cases with milder phenotypes (182).

A single-subject and group-analysis study (183) on patients with ABPE demonstrated that this syndrome is characterized by patterns similar to studies in rolandic epilepsy (focal BOLD signal changes in the spike field) as well as patterns observed in CSWS (distant BOLD signal changes in cortical and subcortical structures), thereby corroborating the concept that idiopathic focal epilepsies of childhood form a spectrum of overlapping syndromes.

An EEG-fMRI study in thirteen patients with ring 20 chromosome syndrome (184) shows specific networks involved by different interictal and ictal events of interest, suggesting that some hemodynamic networks are the expression of epilepsy-related cognitive and behavioral deficits typical of ring 20 chromosome syndrome, whereas others can be common to other syndromes with neurobehavioral regression.

## Intracranial EEG Studies

The indication for video-iEEG monitoring is the absence of a unique focal hypothesis regarding the source of the patient’s seizures (obtained with non-invasive investigation), or the need for cortical mapping of the epileptogenic cortex vs. eloquent cortex (7). Therefore, intracranial electrodes often sample from more than one lobe, although their spatial sampling remains limited and needs to be targeted using all available clinical and paraclinical information. Subdural grids allow dense sampling of the cortical convexity while intracerebral depth electrodes are able to reach deeper structures (e.g., medial temporal structures); combinations of both techniques are feasible. Therefore, iEEG studies represent a unique opportunity to investigate seizure networks in human beings with optimal temporal and excellent spatial resolution.

The concept of the seizure-onset zone as a single, circumscribed brain region implies that, assuming that intracranial electrodes sample this region, ictal iEEG activity should invariably start there. Careful observation of ictal iEEG recordings, however, reveals that this is not always the case. Rather, there are patients in whom clinically indistinguishable seizures seem to start at any of two or more distinct brain areas (100). Observations such as this were one of the major factors spurring the interest in considering seizure-generating brain regions as distributed networks. Therefore, the seizure-onset zone could be seen as the particular regions with the lowest seizure threshold while other regions could also give rise to independent seizure onsets, which explains the need to consider more than the sole seizure-onset zone for estimating the epileptogenic zone. In an attempt to quantitatively analyze seizure-onset patterns, Bartolomei and colleagues (185) developed the epileptogenicity index (EI), which takes into account the transition of iEEG activity toward higher frequencies (a general observation of ictal iEEG patterns) together with the delay in which the transition is observed compared to the ictal onset. This approach has revealed that in a significant portion of TLE patients, the medial and lateral temporal lobe display similar EI, implying that both structures could equally subtend seizure generation. Also of interest, some patients with what seemed like TLE before implantation actually displayed the highest values of EI in the fronto-orbital, opercular, or insular cortex rather than the temporal lobe, and these patients had poorer outcomes after resective surgery, suggesting that they harbored more distributed seizure-generating networks not easily amenable to full resection (186). The number of brain regions with a high EI increases with the duration of epilepsy, suggesting that epilepsy networks may extend over time as a result of plasticity triggered by pathological activity (185, 186).

The same authors analyzed the neurophysiological correlates of alterations of consciousness in TLE (187). They found that alteration of consciousness was associated with increased broadband synchronization in a network of structures, which were mainly extra-temporal, including the thalamus, cingulate cortex, and parieto-temporal association cortex. Consciousness was preserved as long as excessive synchrony was confined to the temporal lobe. Similarly, loss of consciousness in parietal seizures was associated with widespread parietal and frontal synchronization (188). The authors framed these observations into the context of the global workspace theory, in which the sustained synchronization of neuronal activity in widely distributed modules renders perceptions, memories, and intentions available to consciousness (189). This work rejoins observations made with single photon emission computed tomography that temporal lobe seizures causing altered consciousness were associated with widespread cortical and subcortical blood-flow alterations (190). That group later showed increases in the power of delta oscillations in the frontal and parietal association cortices during seizure-related loss of consciousness (191). Results from studies in a rat model of complex partial seizures suggest that these widespread changes are caused by transient alteration of activity in the subcortical septal nuclei (192), implying that the widespread effects of temporal lobe seizures on cortical networks could be mediated indirectly via the midline arousal structures (193).

Measures of directed connectivity in seizure networks are starting to reveal the internal organization of the individual nodes that make up the network. To date, most iEEG studies use mathematical approaches to determine the direction of connections. For instance, using focal cortical dysplasia as a model of a circumscribed seizure-onset zone and applying partial directed coherence analysis, Varotto et al. (12) found that the focal dysplasia indeed acted as the initial generator of ictal activity, as evidenced by its greater out-degree both interictally, pre-ictally, and during ictal onsets [the out-degree is a summary measure of the influence of one network node on all the others (194)]. Cortical-areas remote from the morphological lesion could also be involved in the onset or early propagation of ictal high-frequency activity and could thus represent secondary foci. Wilke et al. (195) used frequency-band-specific betweenness centrality, a graph theoretical measure of the “importance” of a node in network pathways, to demonstrate a significant overlap between the intracranial electrodes showing the highest betweenness centrality and the seizure-onset zone delineated visually by clinical neurophysiologists, as well as the resected cortical area. That correspondence was present both during ictal and interictal recordings and was highest for gamma-band frequencies. In addition, the analysis also revealed nodes with high betweenness centrality that had not been clinically identified as part of the seizure-onset zone. Van Mierlo et al. (196) showed that the single intracranial electrode with the highest out-degree during seizure onsets was included in the clinically defined seizure-onset zone as well as the resection area in all of eight patients. These first findings suggest that analyzing epileptic networks in the framework of graph theory, taking into account the direction of connections between nodes in the network, can help clinicians delineate the primary drivers from secondary nodes in seizure nodes [see also in Ref. (197) for a review]. In the near future, we expect that the tools of graph theory will be applied more generally to iEEG data to describe more fully the spatio-temporal dynamics of seizure networks. Another unique perspective could be offered by the analysis of simultaneous recordings of iEEG and fMRI (198, 199) to combine the spatial resolution of iEEG with the mapping of whole-brain BOLD changes related to epileptic activity. This could allow bridging the poorly understood gap between increased iEEG connectivity and reduced BOLD connectivity within epileptic networks (108).

### 

#### Micro-electrode studies in human beings

Another potential breakthrough in the investigation of epileptic networks could stem, in a somewhat paradoxical fashion, from micro-electrode array recordings, which revealed new insights on the pathophysiology of epilepsy. Schevon et al. (200) inserted arrays comprising of 96 electrodes arranged in a 4-by-4-mm square pattern in the putative seizure-onset zone allowing to record single unit activity in cortical layers 4 and 5 as well as recording the local-field potentials. They showed that there is a sharp delineation (at a sub-millimetric scale) between cortical-areas involved in intense hypersynchronous firing (the hallmark of ictal activity, based on animal studies) and adjacent areas with only mildly increased firing rate and synchrony. Crucially, visual inspection of the iEEG alone did not allow distinguishing between what the authors termed the seizure core and its (presumably) inhibitory penumbra. The same investigators further proposed that ictal high-frequency oscillations phase-locked to the lower-frequency, high-amplitude ictal iEEG recorded by standard intracranial electrodes might represent a signature of increased firing in the seizure core (201). These new findings open the possibility of investigating neuronal firing in distributed seizure networks using conventional iEEG electrodes, without the need for micro-electrode arrays. Future work building on these exciting findings will likely increase our understanding of the ways in which seizures alter normal neuronal firing across the nodes of the involved networks.

#### Direct electrical stimulation studies

**Figure 5:**
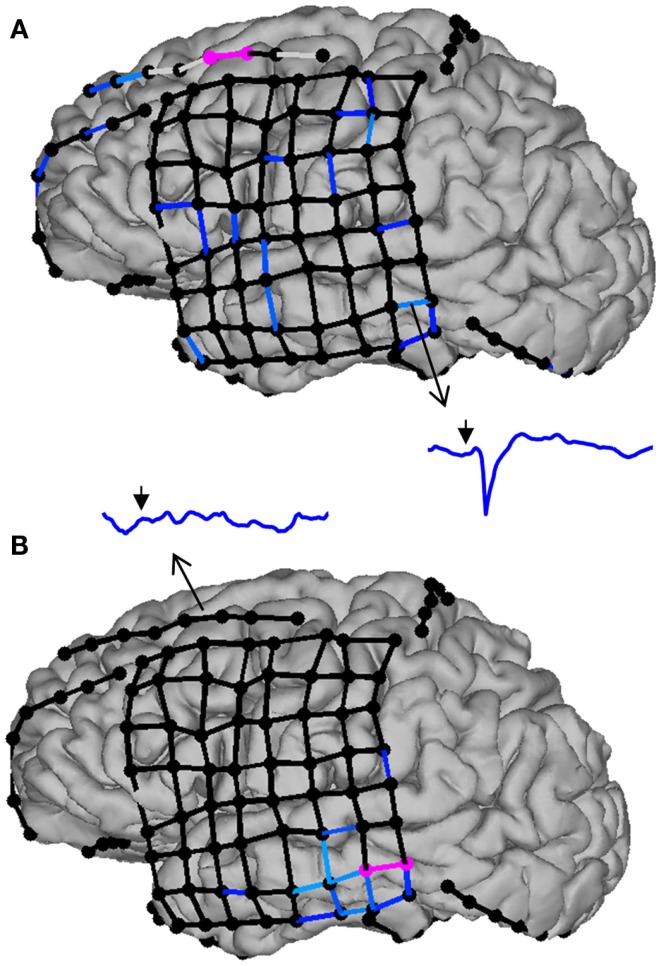
**Evoked effective connectivity reveals the directionality of neural connections in the human brain**. In this example, subdural electrodes are represented by circles, and CCEP responses as lines linking bipolar electrode pairs. Missing (e.g., artifacted) data are indicated by light gray lines, sub-threshold (non-significant) responses by black lines, and significant responses by progressively lighter shades of blue. **(A)** Stimulation of the middle frontal gyrus (electrode pair colored in pink) triggers widespread responses in the frontal and temporal lobes, including the middle temporal gyrus (inset: CCEP waveform from 50 ms before to 250 ms after stimulation; the arrow indicates the time of stimulation). By contrast, stimulation of the middle temporal gyrus **(B)** does not evoke any significant response in the frontal lobe, illustrating that effective connectivity between remote brain structures is not necessarily reciprocal.

Direct electrical stimulation (DES) in epileptic patients consists of administering electrical currents to the brain tissue in order to transiently influence or perturb its function. A technique almost as old as epilepsy surgery, it has mostly been used to probe the function of the cortex directly underlying or surrounding the stimulation site (202–205). In that context, DES is generally delivered at high frequencies (e.g., 50–60 Hz) for a few seconds with the aim of inducing clinical changes in the patient (206). More recently, DES has also been used to investigate FC; in that case, single stimulation pulses are delivered at low frequencies (e.g., 1 Hz) and the readout consists of time-locked perturbations in the activity of points distant from the stimulation site (cortico-cortical evoked potentials, CCEPs) (207). An interesting aspect of DES-based FC assessments is that they are directed, i.e., the effect of stimulation at site A on site B is not necessarily symmetrical with the effect of stimulating B on A (Figure 5). There is an intuitive appeal to this “hands-on” interventional approach to reveal directional connectivity. Evoked effective connectivity was found to correlate with FC measured by resting-state fMRI (22) as well as with anatomical connectivity probed by diffusion tensor imaging (208). It has been pointed out, however, that DES can activate axons in the antidromic as well as the orthodromic direction, and could also stimulate *fibers de passage*, an important caveat to keep in mind when interpreting the directionality information provided by these data (209). This highlights the importance of aiming at obtaining multimodal functional and structural information to better understand brain connectivity and dynamics.

Evoked effective connectivity has revealed strong intralobar connectivity in the temporal and frontal lobes, as well as connections between the frontal and temporal lobes that are more prominent in the frontal-to-temporal than in the temporal-to-frontal direction (210, 211). An intriguing aspect of these studies is the observation that, whereas interhemispheric connections between the frontal lobes are relatively common, temporal-temporal connections appear sparse, being observed in <20% of patients (211). This begs the question of which neuronal pathways are responsible for bitemporal synchronized spiking as well as the propagation of seizures from one temporal lobe to the other one (212). Recently, David et al. (213) generalized this approach offering to develop an atlas of evoked effective connectivity that would eventually allow, through data sharing, sampling most of the human brain’s volume.

Direct electrical stimulation has also been used to specifically evaluate epileptic networks, the general idea being that the responses of remote sites to stimulation of epileptogenic cortex (214) or the responses of epileptogenic cortex to stimulation of remote sites (215) differ from those involving only normal brain tissue. Interestingly, the network of brain areas that respond to DES of the seizure-onset zone overlaps partially but not completely with the areas of ictal propagation, suggesting both that seizures propagate sequentially through multiple nodes in the network and that some existing connections between the seizure-onset zone and distant brain areas “shut down” during seizures (216). Further research combining iEEG and DES, as well as integrating these techniques with fMRI and high-density non-invasive electromagnetic recordings, will improve our understanding of the physiology of seizure networks.

## What We Can Learn from Animal Models

Recording the activity of any node suspected to be determinant in the disease is not feasible in human beings, contrarily to animal research. Moreover, animal-related technologies offer the possibility to desiccate and manipulate cellular and molecular components of such networks, as well as scrutinizing the associated structural and functional alterations. A great perspective in pathological networks study is detecting features associated with the risk of recurrence after a first seizure as well as predicting the evolution toward pharmaco-resistance.

Animal models allow studying networks connectivity and recording the underlying brain activity with high spatial coverage and resolution (217), and addressing the process of epileptogenesis and ictogenesis, including their molecular and genetic mechanisms at cellular and subcellular levels (218–222). Imbalance between excitation and inhibition might not only occur at the local microscopic level (223, 224), but could also reflect dysregulation of excitatory and inhibitory neuronal interactions at a larger (network) scale. Recent evidence emphasizes the modifications of the network dynamic, or network configuration that characterizes, and sometimes precedes or even predicts a seizure. Network analysis could be a powerful tool to more precisely define the different epilepsies and develop new treatments that target networks, instead of focal activity (11, 100).

In animals and human beings, focal onsets have been identified in generalized epilepsy, and complex large-scale network involvement has been shown in focal epilepsies (8, 11, 14). Spontaneous epileptic disease occurs in animals, as in the case of the genetic absence epileptic rats of Strasbourg (GAERS) or in the WAG/Rij rats (225–227); other models studied are epileptic conditions induced by – mainly – chemical or electrical interventions (220). A major animal model of TLE is the kainate, or pilocarpine, model of hippocampal sclerosis (HS) (228–232). Kainate, a glutamatergic agonist, is injected either in the hippocampus or intraperitoneally. It is suspected that the kainate has a tropism for the hippocampus, which led several authors to consider that the kainate induces specifically a HS. Yet, the mechanisms by which kainate induces an epileptic activity is still debated; the immune system and leakage of the blood–brain barrier have been cited as critical for the expression of the disease (233, 234). Hence, it is not excluded that systemic kainate may have diffuse effects on the brain.

Models of focally induced epileptic disorders might avoid this limitation. One of them, electrical kindling, triggers focal epileptic activity using focal electrical stimulation in accordance with standard stimulation parameters, e.g., duration of the stimulation, frequency, and intensity of the stimulus (220, 235, 236). The emergence of a distant pathological activity can be related to remote effects of the focally induced epilepsy, and not to the direct diffuse effects of the electrical or chemical triggers. Electrical stimulation, in particular of the performant-path, has also been described as a model of induced status epilepticus (237, 238).

### Connectivity studies in animals

**Figure 6:**
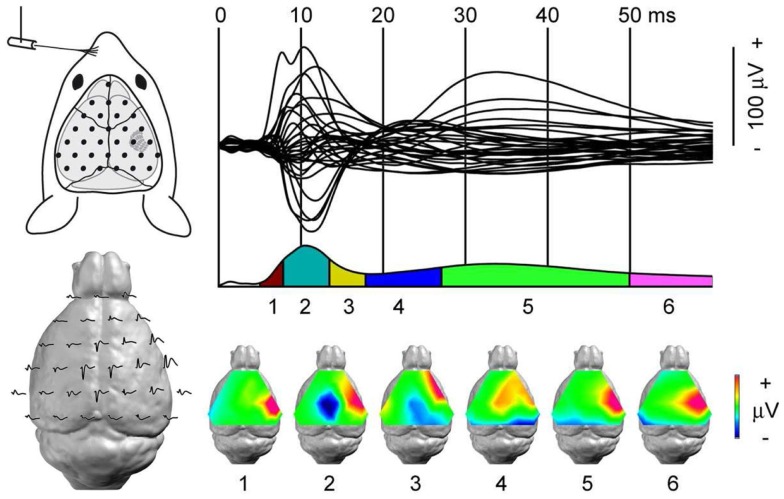
**Dynamic of somato-sensory network mapped with high-density EEG**. Somato-sensory evoked potential (SEP) from left whisker stimulation. *Top left:* each black dot represents the position of one recording electrode; the most anterior one is ground. *Top right:* 31 electrode traces displaying the SEP with sub-milliseconds resolution. *Bottom left:* the same electrode traces represented over the mouse brain. In the lissencephalic mouse brain, dipoles are estimated to be generated below the recording electrodes. *Bottom right: s*egmentation of the SEP in six stable configurations of potential maps. The technique’s high spatial and temporal resolutions identify the first component, somato-sensory barrel field activity, followed by motor cortex and contra-lateral somato-sensory areas recruitment within a few milliseconds. Adapted from Megevand et al. (243) with permission.

Electrophysiology can assess connectivity and RS networks in animal models of epilepsy by recording several brain regions simultaneously. The great advantage is that the signal can be directly linked to neuronal activity. Using intrahippocampal recording in a rat model of induced TLE, Wang et al. (239) showed that neuronal pairs presented a decreased FC prior to the status epilepticus induced by an intraperitoneal injection of pilocarpine. Using Graph Theory measures in an *in vitro* Mg^2+^-free model of hippocampal epilepsy, Gong et al. (240) reported the modifications in network configuration that appear in parallel to epileptiform discharges. More interestingly, they revealed that the changes in network configuration appeared before and lasted longer than the epileptiform discharges (240). These two observations suggest that the classical ictal activity, i.e., the presence of spikes in the EEG, could be the resultant of network reconfiguration, i.e., it could even be an epiphenomenon of a more profound alteration in brain connectivity, indicating that it could be possible to identify certain network alterations as a biomarker of epilepsy. Such studies aimed at identifying markers of an upcoming ictal activity and have mainly looked at the local activity changes (241). Knowledge on remote involvement is sparse. Recent works (224) showed structural alterations remote from the focus, but only a few evidence of distant, abnormal neuronal activity exists (242). Major advancement has been made to record as many neurons or neuronal populations as possible at the same time (145, 243–248); this shows the feasibility to investigate large-scale networks in animal models with high temporal and spatial resolution (Figure 6). Their combination with effective connectivity measures (25) will help to better understand the hierarchical organization of epileptic networks. Gong et al. (240) demonstrated the leading activity of pyramidal cells over granular cells in an *in vitro* model of TLE, illustrating the utility of effective connectivity in the field of epilepsy.

Research on animal models of epilepsy has been dominated by invasive electro-physiology technique. Recently, the combination of EEG and fMRI has emerged with interesting results, such as those reported by Englot et al. (192), where they describe how a partial limbic seizure lead to neocortical slow-wave activity; yet, technical issues makes difficult to obtain combined EEG-fMRI in awake animals. As in Englot et al. (192), fMRI could possibly be a powerful *in vivo* screening method for anatomical regions that could then be more deeply investigated with EEG.

Using fMRI, Mishra et al. (249) showed that rats submitted to traumatic brain injury through left parietal fluid percussion presented a decreased correlation coefficient between the left parietal cortex and other brain regions. Dysfunctional activity in the left parietal cortex, as highlighted by the decreased correlation coefficient could have been expected, yet the pattern of resting BOLD-fMRI connectivity showed that only certain regions were specifically affected, namely the left hippocampus and the contra-lateral parietal cortex. This illustrates that BOLD-fMRI can be used to identify secondary dysfunctional brain regions in rodents following a proepileptogenic injury (249). The same group investigated with fMRI the FC in WAG/Rij rats (250) and found an increased correlation between brain areas suspected to be involved in seizures when compared to non-epileptic rats; more importantly, this increase was observed outside of the ictal periods. Choi et al. (251) performed a FC study using the ^18^fluorodeoxyglucose positron emission tomography (PET) signal in a rat model of TLE. They revealed the decreased correlation of several pairs of brain structures, most of them included left amygdala and left entorhinal cortex (251). Hence, despite the systemic injection of pilocarpine, the affected network appeared to be mainly restricted to the left hemisphere (251). It would have been very interesting to see if the electro-physiological counterpart of such functional deficit was also restricted to one hemisphere, yet no EEG recording was reported. Asymmetry in the central nervous system is well recognized, e.g., asymmetry of the temporal lobes, but the reason why the left hemisphere appears to be more functionally altered in this rat model of TLE is unclear, although electro-physiological experiments suggest that the left hemisphere is indeed more prone to develop epileptic discharges (252). The authors claimed that the PET images were acquired in the interictal period, but no EEG recording was used (251); yet, if true, this would suggest that epileptic animals can be identified as such on the basis of the FC of particular networks outside of any ictal activity. These studies (249–251) indicate that the pathological process in these rats is ongoing: the epileptic brain is not suffering from epilepsy only during seizures.

The anatomical basis of FC is largely unclear. Zhou et al. (253) nicely investigated the anatomical substrate and plasticity of such connections. They observed that after partial posterior callosotomy of wild-type rats, the FC of the auditory and visual cortices decreased at day 7 and returned to baseline at day 28, whereas this decrease was still present in rats submitted to complete callosotomy (253). The authors concluded that it could be possible to identify the anatomical basis of FC, and that these functional connections were also capable of plasticity. This is an important proof-of-concept: it is possible to identify morphological substrate of functional connections and manipulate them.

### Differential involvement of specific brain regions in animal models of generalized epilepsy

Different rat models of generalized absence epilepsy have been studied and all share the presence of the characteristic SWDs (254, 255). Using combined EEG-fMRI in WAG/Rij rats, Mishra et al. (250) demonstrated that during SWDs, the associated fluctuations in the BOLD signal were specific to certain brain regions. Indeed, the somato-sensory barrel field showed an increase, whereas the striatum showed a decrease in the BOLD signal and cerebral blood-flow (225, 250). On the other hand, the local-field potential (LFP) and the multi-unit activity (MUA) were increased in both regions (225). Vascular steal or dopamine-regulated blood volume could account, at least in part, for this lack of matching between BOLD signal and CBF on the one hand and LFP and MUA on the other hand (225). An earlier study using surface and deep EEG recordings in the same rat-model showed that these rats shared a similar focus located in the ventrolateral part of the somato-sensory cortex (SC) (145). More importantly, the authors observed that the ictal activity of the cortical focus preceded the one in the thalamus, suggesting that the cortex was leading the thalamus (145). Nersesyan et al. (256) investigated the relation between SWDs and CBF in the same animal model. They showed that regions involved in SWDs, i.e., SC, presented a 1- to 2-s delayed increase in CBF during a SWD, whereas this increase did not appear in regions not involved in the SWDs, such as primary visual cortex (256). In a parallel work using the same animal model of absence epilepsy, they observed that the BOLD signal was not equally modified across brain regions during a SWD (257): the somato-sensory and motor cortices, as well as subcortical regions, i.e., thalamus, basal ganglia, and brainstem, showed an increased BOLD signal, whereas other regions such as the occipital cortex did not show such a modulation of the signal (257). Again, the increase in the BOLD signal appeared with a lag of a few seconds after the electro-physiological SWDs (257). This finding is in contrast with a work by Desalvo et al. (258), in which they used a rat model of generalized tonico-clonic seizures induced by injection of iv bicuculline, and observed that BOLD increased significantly in primary and secondary somato-sensory cortices, as well as in primary auditory cortex and thalamus *before* the onset of electro-physiological seizures. The role of the SC in initiating GSWDs was further investigated through inactivation of this cortical region in GAERS animals (259). The pharmacological inactivation of the SC with the sodium channel blocker tetrodotoxine prevented the spike-and-wave activity; yet unilateral application of the drug did not completely abolish the abnormal contra-lateral oscillations. On the whole, these studies highlight the importance of abnormal focal brain activity as a potential trigger of generalized seizures (258). The identification of interacting yet independent nodes within a network of suspected generalized epilepsy is a major advance in epilepsy research. Indeed, it will permit to refine the therapeutic intervention toward the manipulation of one particular and decisive node.

### Short-range and long-range network modulations in animal models of focal epilepsy

Different animal models of focal epilepsy exist (220), such as the kainate- or pilocarpine-models of MTLE (228, 229, 260), posttraumatic epilepsy (261, 262), or electrical kindling (227, 263). Despite an initially focal insult, recent evidence (e.g., 242) shows that remote brain areas become affected by the pathological activity of the epileptic focus.

The induction of a focal epileptic syndrome in a rat model of generalized epilepsy allows better understanding how these two entities interact. Carcak et al. (227) took advantage of the fact that absence epilepsy may increase the resistance to limbic seizures. They investigated the role of the cortico-thalamo-cortical circuitry, involved in SWDs, in the development of limbic seizures induced by unilateral electrical stimulation of the rat amygdala. Whereas control rats, i.e., those without absence epilepsy, presented all convulsive epileptic seizures following amygdala electrical stimulation, rats suffering from absence epilepsy did not (227). In order to understand how the circuit involved in absence epilepsy could affect the one of TLE, the authors investigated the spontaneous activity in the reticular thalamic nucleus (RTN), known to be involved in the slow-waves discharges that characterize absence epilepsy (227). Remarkably, the electrical stimulation of the amygdala had a different effect on the mean firing frequency of neurons of the RTN: in not-stimulated animals, there was no significant difference between epileptic and non-epileptic rats, whereas the increase after stimulation was higher in epileptic rats when compared to non-epileptic rats (227). This suggests first that the development of an epileptic focus alters the activity of neurons in the RTN and second that this alteration depends on the activity before the induction of the epilepsy. The use of Wistar rats as controls for GAERS rats in that study is commonly accepted, but could still be questioned; yet the conclusion would still remains the same: the effects of an epileptic focus seem to depend on the brain state in which it is being established. It would hence be interesting to investigate how an epileptic focus affects a given network, but also how a particular network configuration can modulate the effects of an epileptic focus. The involvement of the thalamus in propagation of temporal lobe seizures has already been the scope of several studies (156, 264). If the thalamus has a major role in the generation of SWDs (227), this could highlight the relevance of studying the interaction between hippocampus and thalamus, in the context of focal epilepsies.

Hippocampal sclerosis is a frequent lesion that has been deeply investigated, although, most of the works conducted local, intrahippocampal recordings. Yet, recent publications have shown the involvement of remote brain areas. Using 16 bipolar deep electrodes in the pilocarpine rat-model of HS, Toyoda et al. (247) showed that the initial focus varied from one seizure to another in each individual rat. The subiculum, the dorsal and ventral hippocampus, and the amygdala were the regions where seizure onsets were most often recorded. All regions could be considered as belonging to the same network; indeed, an interesting observation is that most seizures were convulsive, and this did not depend upon where the seizure started (247). This suggests that the involved network is more determining than the seizure-onset zone for the clinical expression of a seizure. Long-range modifications in the kainate mouse-model of TLE were also observed. It has been shown that non-injected hippocampus presented indeed morphological alterations, notably in the expression of the neuropeptide-Y, which is known to modulate neuronal activity (265, 266), and electro-physiological changes, such as significant decrease in the power of the theta frequency band (265). *In vitro*, Khalilov et al. (267) demonstrated that a mirror focus in the contra-lateral hippocampus appears after 10–15 successive applications of kainate in the ipsilateral hippocampus. These findings are in line with the hypothesis that an epileptic focus leads to permanent electro-physiological and morphological modifications in remote brain areas (268–270). Other works have also stressed the possibility that subcortical brain regions, such as the basal ganglia, could influence or even inhibit the progression of an ictal activity originating from the temporal lobe (271, 272).

Evidence of distant brain involvement arises also from electrically induced epilepsy. For instance, during hippocampal seizures induced by electrical stimulation in rats, the frontal neocortex presented a parallel modification in spontaneous activity, i.e., fast polyspike activity when the seizure was generalized and slow oscillations when it was partial (242). This example illustrates that distant brain areas are affected even after a few or only one focal epileptic seizure. It would be extremely interesting to study how this involvement evolves in a chronic disease.

On the whole, evidence exists that other brain areas are recruited in propagation or in inhibition of the seizure spread. Hence, the epileptic threshold does not seem to depend only on the imbalance between excitation and inhibition within the focus, but could also be determined by the intricate interactions between the components of a given network.

### Experimental therapeutic interventions on the epileptic network

Conceiving epilepsy as a network disease has therapeutic consequences. The classical view is to modulate the activity of the so-called epileptic focus, or seizure-onset zone, in order to control the disease. Yet, any node of an epileptic network could possibly be a target. In this sense, open-loop or closed-loop devices, either through electrical or optogenetic stimulation, are promising tools for generalized (217) as well as for focal epilepsy (273, 274). Major work has shown that it is possible to identify critical nodes in a given epileptic network: the modification of their activation – mainly inhibition – could help to control, or even stop an ictal activity. Paz et al. (274) showed in a rat model of cortical epilepsy that the inactivation through optogenetics of the thalamic ventrobasal nucleus could stop an ongoing seizure. In the same line, Langlois et al. (264) showed in an *in vivo* model of TLE that DBS of the ipsilateral parafascicular nucleus of the thalamus (PF) stopped the ongoing hippocampal paroxysmal discharges (HPD), while higher current intensities were needed to stop the HPD if DBS was applied to neighboring areas (264), illustrating the specificity of PF in controlling HPDs. The anterior thalamic nucleus (ANT) appears also to be involved in control or spread of epileptic activity of mesial temporal onset (156). Ablation or electrical stimulation of ANT increases the epileptic threshold (263, 275–277); yet, opposite results have also been observed (278). On the whole, ANT is a recognized target for refractory epilepsy, although mechanisms by which manipulation of the ANT increases epileptic threshold are poorly understood. Use of animal research and the possibility to identify how the activity of ANT may modulate epileptic activity at remote sites, e.g., with the use of effective connectivity measures, is crucial to tailor therapeutic interventions. Such recent evidence shows that the manipulation of the primary epileptic focus does not seem to be the only possibility to achieve the control of an epileptic disease. The thalamus in particular, and other subcortical regions as well (272) have been identified as major targets for epileptic network modulation culminating in clinical applications in the form of DBS of ANT in focal epilepsies (279).

## Conclusion

With increasingly complex methodological strategies and an ever-increasing wealth of possible approaches, the study of brain connectivity and its neuroscientific and clinical correlates are very promising. Nevertheless, the application of connectivity techniques for diagnostic or prognostic purposes requires further studies to be firmly grounded by invasive studies and sufficient follow-up investigations before it can be reliably applied to the clinical management of individual patients. Combining functional techniques can lead to the achievement of complementary information for improving each single technique.

Focal epilepsies, despite focal epileptogenic zone, are diseases affecting the whole brain: altered large-scale FC is reflected in neuropsychological features of individual specific syndrome. Hippocrates (400 years b.c.) considered epilepsy as a systemic disease, centered in the brain, due to an altered “defluxion of cold phlegm” through the body. In more recent times, the concept of epilepsy as “focus disease” has been largely developed (280–282), whereas in the last decade it has shifted to a “brain-network disease” (15). In parallel to the “brain-network” concept of epilepsy, psychiatric and neurological co-morbidities, such as strokes, dementia, and migraine are more and more defined. Interestingly, somatic co-morbidities have also come to light, since several medical conditions, such as cardiac, gastrointestinal, and respiratory disorders, are often associated with epilepsy (283). These findings may lead to re-consider epilepsy as a “systemic disease,” this time with the diagnostic and therapeutic knowledge obtained recently by ground-breaking work on network analysis.

Concerning “generalized” epilepsy, neuro-imaging, and especially connectivity studies have allowed considering them as focal brain disorders with fast bilateral discharge propagation. This concept leads to the idea that focal and generalized epilepsies are the two extremes of a single spectrum and to a possible new way of studying mechanisms of AED: do they have an effect on particular nodes of a network where receptors are more expressed? Is it possible to detect an anatomical target to avoid generation/propagation of seizures, using disconnection or stimulation? For all these reasons, translational research in light of network analysis, based on fundamental science through animal experiments and clinical perspectives through human research, opens new opportunities to better understand the complexity of epilepsy and define new and more effective treatments for patients.

## Conflict of Interest Statement

The authors declare that the research was conducted in the absence of any commercial or financial relationships that could be construed as a potential conflict of interest.
